# Normalizing suffering: A meta-synthesis of experiences of and perspectives on pain and pain management in nursing homes

**DOI:** 10.3402/qhw.v11.31203

**Published:** 2016-05-11

**Authors:** Mojtaba Vaismoradi, Lisa Skär, Siv Söderberg, Terese E. Bondas

**Affiliations:** 1 Faculty of Professional Studies, Nord University, Bodø, Norway; 2 Department of Health, Blekinge Institute of Technology, Karlskrona, Sweden; 3 Department of Nursing Sciences, Mid Sweden University, Östersund, Sweden

**Keywords:** Older people, nursing homes, meta-synthesis, meta-ethnography, pain, pain management, suffering

## Abstract

Older people who live in nursing homes commonly suffer from pain. Therefore, relieving suffering among older people that stems from pain demands knowledge improvement through an integration of international knowledge. This study aimed to integrate current international findings and strengthen the understanding of older people's experiences of and perspectives on pain and pain management in nursing homes. A meta-synthesis study using Noblit and Hare's interpretative meta-ethnography approach was conducted. Empirical research papers from journals were collected from various databases. The search process and appraisal determined six articles for inclusion. Two studies were conducted in the US and one each in Iceland, Norway, the UK, and Australia. The older people's experiences of pain as well as perspectives on pain management from all involved (older people, their family members, and healthcare staff) were integrated into a theoretical model using three themes of “identity of pain,” “recognition of pain,” and “response to pain.” The metaphor of “normalizing suffering” was devised to illustrate the meaning of pain experiences and pain management in nursing homes. Society's common attitude that pain is unavoidable and therefore acceptable in old age in society—among older people themselves as well as those who are responsible for reporting, acknowledging, and relieving pain—must change. The article emphasizes that pain as a primary source of suffering can be relieved, provided that older people are encouraged to report their pain. In addition, healthcare staff require sufficient training to take a person-centered approach towards assessment and management of pain that considers all elements of pain.

Declining physical functioning and an inability to rely on family members to maintain self-care (Harrefors, Sävenstedt, & Axelsson, 2009; Nakrem, Vinsnes, Harkless, Paulsen, & Seim, 2013) may lead many older people to the option of a nursing home care. The number of nursing home residents is increasing, making it increasingly difficult to provide high-quality pain management (Habjanic, Saarnio, Elo, Turk, & Isola, 2012).

Moving into a nursing home has been described as an overwhelming life change for many older adults (Brandburg, Symes, Mastel-Smith, Hersch, & Walsh, 2013). Previous research has reported that older nursing home residents commonly suffer from inappropriate pain management as an everyday experience (Gran, Festvåg, & Landmark, 2010; Takai, Yamamoto-Mitani, Okamoto, Koyama, & Honda, 2010), which can have serious consequences on their health (Higgins, Madjar, & Walton, 2004). Pain has been described as an important and neglected healthcare issue in the nursing home context (Tse & Ho, 2014). Pain can hinder older people's daily life activities and reduce their desire to participate in social activities (Tse, Leung, & Ho, 2012). A nursing home should be a place of safety and peace for older people (Andersson, Hallberg, & Edberg, 2008; Payne & Fletcher, 2005). An important component of high-quality nursing care is that it provides situations in which residents feel comfortable (Fjær & Vabø, 2013).

Pain may lead to isolation and feelings of loneliness (Gran et al., 2010). Pain may also be associated with depression as well as sleep and overall function disturbances (Blomqvist & Edberg, 2002; Monroe et al., 2014; Ross & Crook, 1998). Suffering from untreated pain can lead to greater cognitive impairment and functional loss (Monroe et al., 2014). Pain contributes to reduced dignity and restricted life space, influencing older people's overall psychological well-being (Blomqvist & Edberg, 2002; King et al., 2012). According to Pleschberger (2007), the main quality of a dignified death is a lack of pain and suffering related to pain.

The lack of quality pain management has led to the view of pain as an expected experience in nursing homes (Higgins, 2005; Higgins et al., 2004). Suffering related to pain among older people demands that healthcare professionals collaborate to improve the quality of care delivered to older people (Teno, Kabumoto, Wetle, Roy, & Mor, 2004). Achieving optimal pain management in nursing homes continues to be a challenge for all healthcare professionals. Moreover, both a lack of sufficient knowledge and misconceptions about pain in nursing homes contribute to unsafe, inadequate, and inappropriate pain management (Clark, Fink, Pennington, & Jones, 2006; Tse & Ho, 2014). Deeper understanding of and knowledge about pain is required to design and implement interventions that will lead to reduced prevalence of pain in nursing homes (Dobbs, Baker, Carrion, Vongxaiburana & Hyer, 2014; Jones et al., 2004).

Several qualitative studies have investigated the phenomenon of pain in nursing homes from the perspectives of different stakeholders and in various contexts. However, no meta-synthesis was discovered that could guide evidence-based practice. Moreover, the literature lacks integrated and interpreted knowledge of pain and pain management from the perspectives of older people and others involved in pain management in nursing homes. Therefore, this meta-synthesis study aims to integrate current qualitative international findings and enhance the understanding of experiences of and perspectives on pain and pain management in the context of nursing homes.

## Materials and methods

### Design

A meta-synthesis study using Noblit and Hare's (1988) interpretative meta-ethnography approach was chosen. The characteristics of meta-synthesis comprise interpretation, integration of qualitative research findings about a particular phenomenon, consideration of variations, and advancement of a theoretical structure for future-related studies (Bondas & Hall, 2007a, b; Sandelowski & Barroso, 2007). Accordingly, this meta-synthesis study was conducted by following the steps below:

- deciding on the phenomenon and the relevance of studies for synthesis;
- searching for and reading the studies;
- determining the relevance of the studies by comparing the various studies’ findings to discover whether their relationships were reciprocal (similar or analogous), refutational (in opposition), or cumulative (in a line of argument);
- translating the studies into one another by developing themes in a way that sought to preserve themes and conserving unity;
- synthesizing this translation and creating a whole greater than its individual parts;
- reporting the synthesis, providing a coherent description of the study phenomenon, and creating a meta-finding (Noblit & Hare, 1988; Polit & Beck, 2008).

### Search strategy and criteria

A precise systematic search for and appraisal of articles related to the study topic were conducted. Before initiating the search process, consultation with a librarian, a pilot test in electronic databases, and the authors’ experiences of key terms commonly used in the international literature helped the researchers identify the review question and keywords. The search process took as its subject relevant empirical research papers that had been published in journals, without any year limitation, found in online databases including: PubMed (including Medline), CINAHL, SCOPUS, OVID, Wiley Online Library, and Science Direct. The applied key search terms were: “elderly” and “nursing home” combined with “pain” and “qualitative” in any part of the articles. In line with ancestry search and in order to maximize coverage, back and forward tracking of references as well as manual abstract searches were conducted from the reviewed articles. The search strategy and the results of each search step are presented in Table I.

**Table I:** The search strategy and results of different phases of the meta-synthesis process.

Years	Database	Total	Selections based on title	Selections based on abstract	Selections based on full text and inclusion criteria
All years	Cinahl	18	3	1	0
	Scopus	486	16	2	0
	PubMed (including Medline)	79	10	0	0
	Ovid	1872	15	5	1
	Wiley Online Library	2715	24	14	5
	Science Direct	38	5	1	0
	Manual search/backtracking references	125	8	0	0
	Total	5333	81	23	6

The inclusion criteria for the studies were (1) peer-reviewed empirical qualitative studies in the healthcare disciplines, (2) studies that focused on perspectives on and experiences of pain and pain management among older people in nursing homes, (3) studies conducted with older people with intact or sufficiently intact cognitive status, and (4) studies published in English in online scientific journals. The exclusion criteria were studies conducted on the experiences of older people in healthcare settings other than nursing homes and studies with mixed-methods design for which the separation of qualitative and quantitative findings was impossible.

### Search process and quality appraisal of studies

Each author conducted all phases of the search process independently. The authors held frequent discussions throughout the study to reach agreement on the search process. The thorough literature search performed using the key terms produced 5333 articles. Title selection using the inclusion criteria and the deletion of duplicate titles reduced this number to 81 articles. The abstracts of the 81 articles were then checked against the aforementioned inclusion criteria, and 23 articles were subsequently selected. The full texts of these 23 articles were obtained and checked for quality using the Critical Appraisal Skills Programme Qualitative Research Checklist (CASP, 2013). Not every item had the same importance for the article's quality; therefore, no scoring system was used for quality appraisal. As a result, the authors held frequent discussions about the focus of the articles and the main methodological issues that would damage the quality of findings. In the end, six articles were included based on their high degrees of all appraisal domains including aim and scope, design, analysis process, findings, relevance, and transferability.

### Synthesis process

The authors followed the interpretative process based on the approach outlined by Noblit and Hare (1988). The four members of the research team independently read and reread the six articles that were included and created and compared a list of findings from each study (metaphors, themes, and categories). An initial assumption about the relationship between the studies was made. The studies were set against each other since they represented the different perspectives of older people, caregivers, family members, and significant others. The authors worked separately to follow Noblit and Hare's (1988) analysis process of “translating the studies into one another” (p. 28) and subsequently discussed their different understandings, which were enhanced by their different cultural and scientific backgrounds. The authors found that the studies were analogous and could be analyzed as reciprocal. Finally, they agreed on an overarching metaphor and theoretical model that was constructed based on the developed themes and subthemes. In line with interpretation, the researchers provided a coherent description and explanation of the study phenomenon by connecting the findings of their individual research reports to create one meta-finding.

Meta-synthesis can be criticized for removing the original studies’ findings and stripping them of their contexts (Sandelowski & Barroso, 2007). However, the validity of this meta-synthesis rests in its interpretive logic, where the findings of the research reports were reframed and exhibited in the study final product (Sandelowski & Barroso, 2007). This process provided an audit trail for readers by describing the analysis process in detail, by conducting a thorough literature search, and by precisely appraising the reviewed articles (Bondas & Hall, 2007a, b).

## Results

The studies included in this meta-synthesis are summarized in Table II. The studies were published between 1995 and 2010. Two of the studies were conducted in the US (Clark, Jones, & Pennington, 2004; Mentes, Teer, & Cadogan, 2004), and one study each was conducted in Iceland (Gudmannsdottir & Halldorsdottir, 2009), Norway (Gran et al., 2010), the UK (Higgins, 2005), and Australia (Yates, Fentunan, & Dewar, 1995). The methodologies of the studies included ethnography (Clark et al., 2004), phenomenology (Gudmannsdottir & Halldorsdottir, 2009; Higgins, 2005), and other types of qualitative descriptive and interpretive analysis (Gran et al., 2010; Mentes et al., 2004; Yates et al., 1995). The studies mainly focused on pain assessment and expression (Clark et al., 2004; Mentes et al., 2004; Yates et al., 1995), chronic pain (Gudmannsdottir & Halldorsdottir, 2009; Higgins, 2005), and the influence of pain on daily life (Gran et al., 2010). Although Clark et al. (2004) did not directly specify their study's total number of participants, the study participants consisted of 109 people (both male and female) including 82 residents, 16 family members and significant others, and 11 formal caregivers from 28 nursing homes.

**Table II:** Characteristics of the studies selected for meta-synthesis.

Author(s), year, country	Aim	Methods	Sample and setting
Clark et al., 2004, USA	To describe the kinds of pain assessments nursing home staff use with nursing home residents and the characteristics and behaviors of residents that staff consider as they assess pain	Ethnography	21 focus groups with the range of 6–12 licensed and unlicensed staff from 12 nursing homes
Gran et al., 2010, Norway	To gain an understanding of how nursing home residents experienced pain and how it influenced their lives and daily living	Kvale's method of interpretive analysis	15 residents from three nursing homes
Gudmannsdottir & Halldorsdottir, 2009, Iceland	To examine the essentials of the experience of residents in chronic pain in nursing homes	Interpretive phenomenology	12 residents from three nursing homes
Higgins, 2005, UK	To explore the experience of being old and in chronic pain while living in a nursing home	Merleau-Ponty's phenomenology	13 residents from three nursing homes
Mentes et al., 2004, USA	To gather and evaluate whether information from family members and friends about a patient's lifelong pain behavior and expression improves pain detection in cognitively impaired residents and to evaluate pain information from formal direct caregivers who cared for the relatives of these family members	Content analysis	14 family members and 2 significant others of the 20 NH residents and 11 formal caregivers of two nursing homes
Yates et al. 1995, Australia	To investigate the views of pain and pain management practices held by elderly people living in long-term residential care settings	Qualitative analysis	42 residents from five residential care settings

### Perspectives on and experiences of pain and pain management in nursing homes

**Figure 1.**
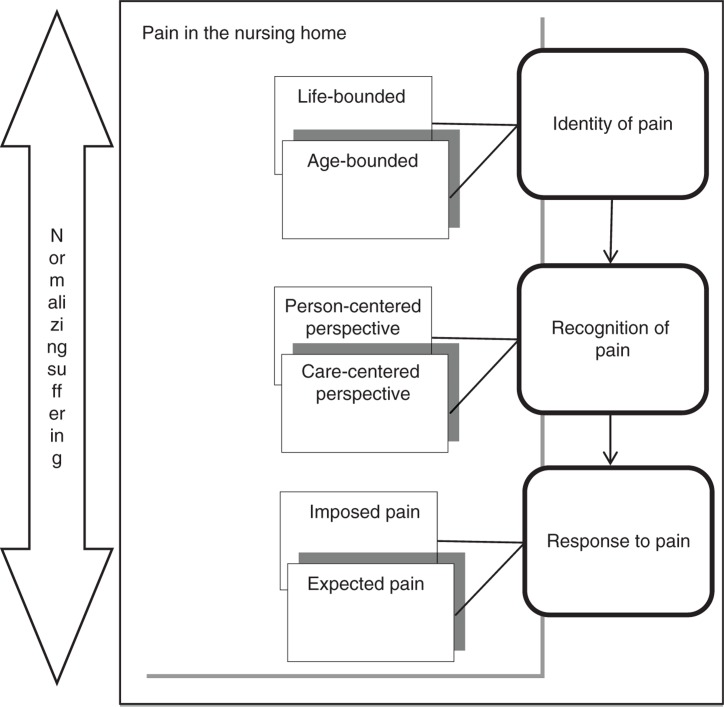
Theoretical model of experiences of and perspectives on pain of elderly people in the nursing home.

Older people's experiences with pain in nursing homes, based on the perspectives and experiences of all involved in pain management, were integrated using three themes: “identity of pain,” “recognition of pain,” and “response to pain.” These themes integrated the experiences of the participants of the meaning of pain, how pain was experienced, and how it was managed in nursing homes. Each theme comprised two subthemes. In line with the meta-synthesis’ aims to integrate current international findings and to present an interpretation of experiences of and perspectives on pain in the nursing home context, the metaphor of “normalizing suffering” emerged in this study. A theoretical model integrated and summarized the main features of the findings (Fig. 1). The metaphor was described further using the three themes and six subthemes (Table III). The meaning of the themes and subthemes as well as an overview of the metaphor that integrates the study findings are described as follows.

**Table III:** Themes and subthemes developed in the process of meta-synthesis to integrate the findings.

	Themes	Meaning	Subthemes	Key aspects and their sources	Articles
Metaphor (normalizing suffering)	Identity of pain	Quality of pain, sources of pain, factors affecting pain, predictability of pain, consequences of pain	Age-bounded	Chronic problems (O, F), multiple physical pain (O, F), episodes of illness (O), iatrogenic pain (O, S)	Gran et al. (2010), Gudmannsdottir and Halldorsdottir (2009), Higgins (2005), Mentes et al. (2004), and Yates et al. (1995)
			Life-bounded	Mood and emotion changes (O, S), huge losses in life (O), losing independence (O), being abandoned (O), losing freedom (O), being pressurized (O), sadness and loneliness (O, S)	Clark et al. (2004), Gran et al. (2010), and Gudmannsdottir and Halldorsdottir (2009)
	Recognition of pain	Validation of pain, vulnerability and uncertainty, taking pain seriously, reporting of pain, being noticed by professional eyes	Care-centered perspective	Dramatic description of pain (F), lack of trust to reported pain (O), ambiguous portrayal of pain (F, S), overlap with other feelings (F, S), denial or exaggeration of pain (F, S), attitudinal barrier to assess pain (O, S)	Clark et al. (2004), Gran et al. (2010), Gudmannsdottir and Halldorsdottir (2009), Higgins (2005), Mentes et al. (2004), Yates et al. (1995)
			Person-centered perspective	Language insufficiency to describe pain (O, S), cognitive impairments (F, S), communication barriers (S), non-formal assessment (S)	Clark et al. (2004), Higgins (2005), and Mentes et al. (2004)
					
	Response to pain	Pain relieving methods, coping with pain, attitudes and expectations of pain-free living in the old age, living conditions in the nursing home, caring about	Imposed pain	Aging is not necessarily mean pain (O), non-pharmacologic pain relief strategies (O, F, S)	Gran et al. (2010), Gudmannsdottir and Halldorsdottir (2009), Higgins (2005), Mentes et al. (2004), and Yates et al. (1995)
		pain by healthcare staff, available methods of pain relief	Expected pain	Pain as an inseparable part of living (O, F, S), putting up with pain (O), pain as a sign of courage (O), testing faith and higher spirituality (O), being afraid of addiction (O, S), attitudes of pain medication (O, S)	Clark et al. (2004), Gran et al. (2010), Gudmannsdottir and Halldorsdottir (2009), Higgins (2005), Mentes et al. (2004), and Yates et al. (1995)

O, Older people; F, family; S, staff.

#### Identity of pain

Responses to the question of what pain is from older people, caregivers, and significant others in the nursing home context led to an overall, comprehensive picture of pain in terms of “quality of pain,” “sources of pain,” “factors influencing pain,” “predictability of pain,” and “consequences of pain.” However, the descriptions of pain that were provided went beyond its “intensity,” “location,” and “duration.” These descriptions could be divided into the dual identities of “age-bounded” and “life-bounded,” the subthemes of this theme. While the former is a well-known origin of pain as experienced by older people, the latter was described as possessing a separate identity or causing intensified pain that differentiates it from the former (Clark et al., 2004; Gran et al., 2010; Gudmannsdottir & Halldorsdottir, 2009; Higgins, 2005; Mentes et al., 2004; Yates et al., 1995).

Age-bounded

This subtheme relates to the common physical nature of pain that accompanies old age. Localization of pain in various parts of the body (primarily in the joints, muscles, and connective tissues) constituted the older people's main complaints of pain. Chronic problems such as arthritis, osteoporosis, and vascular diseases, which varied widely in nature, intensity, location, and temporality, were the main aspects of pain in the participants’ comments. The research emphasized that pain could be more or less disturbing but nevertheless always present (Clark et al., 2004; Gran et al., 2010; Gudmannsdottir & Halldorsdottir, 2009; Higgins, 2005; Mentes et al., 2004; Yates et al., 1995).

My hip hurts, and also my back. If I sit for a long time, the pain gets worse (Gran et al., 2010, p. 28).

My arthritis, I got it right from my head right to my toes, every joint to everywhere (Yates et al., 1995, p. 670).

Family members of older people also reported an association between painful experiences in nursing homes and health-related issues such as stroke, arthritis, lower extremity amputations, fractures, osteoporosis, pressure ulcers, and diabetes. Older people confirmed the healthcare providers’ perspective that the iatrogenic pain of being moved and manipulated further added to pain from chronic physical diseases (Higgins, 2005; Mentes et al., 2004).

I think she's in pain and can't express it. When I reposition her, she says ‘thank you.’ I know she is having pain in the back or butt and couldn't tell me (Mentes et al., 2004, p. 123).

It just makes up its own mind when to go … I feel like a cry baby but I couldn't bear the nurses to touch me you know. I say “don't grab hold of me there, grab me here” (Higgins, 2005, p. 375).

Life-bounded

The participants’ perspectives on and experiences of pain in nursing homes described the influence of older people's emotions and mood on their experience of pain and its non-physical nature. While feelings and emotions were viewed as causing pain by themselves, they were also intertwined with physical pain or at least as worsening it. In other words, major life changes such as deteriorating health conditions, decreased freedom and independence, the death of loved ones, and the experience of being abandoned and pressured were causes of pain or worsened pain (Clark et al., 2004; Gran et al., 2010; Gudmannsdottir & Halldorsdottir, 2009; Mentes et al., 2004).

[She] complained of achy bone pain. General pain … never really specific. She is a great attention getter (Mentes et al., 2004, p. 121).

Healthcare staff were also curious about older people's pain and suggested an overlap between pain and unpleasant emotions such as sadness, loneliness, and depression (Clark et al., 2004; Mentes et al., 2004).

The face–you can just tell; it changes. You see a sad or changed expression (Mentes et al., 2004, p. 122).

… can cause as much suffering as physical pain. What, then, is pain? And can it be quantified or even identified as a pure sensation? (Clark et al., 2004, p. 740).

#### Recognition of pain

The assessment of pain in the nursing home context was viewed as difficult and permeated with uncertainties. The various perspectives on and experiences of pain could be summarized to highlight the need for a method of pain validation, for taking pain seriously, and for healthcare professionals to notice pain. Consideration of the complexities of pain assessment, the applicability and limitations of routine assessment methods, and the need for innovative ways to assess pain and reduce older people's suffering led to the development of two subthemes: a “care-centered perspective” and a “person-centered perspective” (Clark et al., 2004; Gran et al., 2010; Gudmannsdottir & Halldorsdottir, 2009; Higgins, 2005; Mentes et al., 2004; Yates et al., 1995).

Care-centered perspective

Older people, in general, voted against the effectiveness of routines and organized methods of pain assessment in the nursing home context. When older people's pain was not validated by their healthcare staff, the older people's feelings that they lacked safety and that they were not taken seriously as well as their uncertainty regarding interventions conducted for pain management were enhanced (Gran et al., 2010; Gudmannsdottir & Halldorsdottir, 2009; Higgins, 2005; Yates et al., 1995).

I kept telling her about this ugly pain. She'd take no notice of me. She'd shrug her shoulders and say, “it was arthritis, you have to put up with it” (Higgins, 2005, p. 374).

They're always short of time. Yes all I can say is it would be nice if they had a little more patience. Not that they haven't, but a little more, that's all, to listen to you (Yates et al., 1995, p. 672).

These feelings were supported and validated by comments from family members and healthcare staff. They cited a lack of trust in the common methods used for pain assessment as an attitudinal barrier to pain management, due to exaggerated behaviors and the deficiency of common pain assessment tools (Clark et al., 2004; Mentes et al., 2004).

She was always a real drama queen. She dramatizes her pain when we are with her but is okay when we are gone (Mentes et al., 2004, p. 121).

We did have a pain assessment form that we would do on admission, and then we were supposed to follow up 7 days later. Well, the 7 days later was never being done … that's not necessary (Clark et al., 2004, p. 741).

In response, older people denied their pain and were reluctant to report pain to others in order not to worry others and to avoid being perceived as complainers. The participants commonly mentioned putting up with pain to describe the experience of pain and the pursuit of relief from pain (Gudmannsdottir & Halldorsdottir, 2009; Higgins, 2005; Yates et al., 1995).

Well I think they have enough to do. I don't feel they think I'm invalid enough to be looked after, you know what I mean they can't tell how much pain you're in, so I just don't like to annoy them at all (Yates et al., 1995, p. 672).

I haven't had an answer yet about the pills. Oh well, that's how it is. Nothing you can do about it. You have to take it as it comes. One has become old too–can expect everything (Gudmannsdottir & Halldorsdottir, 2009, p. 323).

Person-centered perspective

Cultural attitudes about pain, communication barriers, and cognitive issues hindered the application of pain assessment scales in the nursing home context. Both older people and healthcare staff believed that the inadequacy of language and beliefs about reporting pain hindered appropriate recognition of pain (Clark et al., 2004; Higgins, 2005; Mentes et al., 2004).

[Pain was] “more than a real ache or more than a discomfort, real big, or real pain falls between the cracks of description” (Higgins, 2005, p. 374).

The staff members experienced frustration with the residents’ belief that they “didn't want to bother anyone” by reporting their pain (Clark et al., 2004, p. 744).

To address the issue of pain assessment, healthcare staff sought informal methods to follow and detect signs of pain (Clark et al., 2004; Mentes et al., 2004). An individualistic and unique method of pain assessment derived from knowing the resident in terms of non-verbal clues of pain and behavioral changes could reveal the possibility of pain in the nursing home context (Clark et al., 2004; Mentes et al., 2004).

… she often rubs her legs, but remember, she is very quiet and won't tell you unless you ask (Mentes et al., 2004, p. 122).

“Know your patient well” or “Nursing staff should pay more attention to patients.… see beyond the body and really look at them every time [staff members] go into the room” (Mentes et al., 2004, p. 122).

#### Response to pain

Older people's reaction to the experience of pain in the nursing home context was influenced by pain's meaning to them, including their attitudes towards and expectations about health levels and the possibility of living pain-free in old age. Living conditions in the nursing home, healthcare staff's concerns about pain, and the available methods of pain relief shaped the meaning of pain for older people and their chosen approach to pain management (Clark et al., 2004; Gran et al., 2010; Gudmannsdottir & Halldorsdottir, 2009; Higgins, 2005; Mentes et al., 2004; Yates et al., 1995). In accordance with the above-mentioned factors, two main themes representing two opposite approaches to pain management were articulated: “imposed pain” and “expected pain.”

####  Imposed pain

Some older people formed their response to pain based on the idea that pain was unwanted and could be avoided. Therefore, when such older people experienced pain, they actively sought help, struggled against the pain, and sought relief from healthcare staff (Gran et al., 2010; Gudmannsdottir & Halldorsdottir, 2009; Higgins, 2005; Mentes et al., 2004; Yates et al., 1995). Non-pharmacologic pain relief strategies such as physiotherapy, massage and touch therapy, and technical aids were some of the treatments delivered to alleviate pain ( Gran et al., 2010; Gudmannsdottir & Halldorsdottir, 2009; Higgins, 2005; Mentes et al., 2004; Yates et al., 1995).

It means a lot what kind of chair I sit in. I found one chair which was really comfortable because it formed around the body (Gran et al., 2010, p. 28).

Well I'm a great believer in rubbing, only I say when you live alone you don't get rubbed often enough I think you would want to be rubbed about three times a day not just put on I think it had to be rubbed and rubbed in (Yates et al., 1995, p. 771).

Both older people and healthcare staff appreciated distraction techniques and positive feedback from healthcare staff as suitable methods for pain relief (Gran et al., 2010; Gudmannsdottir & Halldorsdottir, 2009; Mentes et al., 2004; Yates et al., 1995).

My best cure is opening a book I'm reading and reading bits of the book and that takes a lot of the pain away (Yates et al., 1995, p. 671).

I have so many things to appreciate in my life. I learned in a book once, telling me to do as the “Sun God”—just think about the bright side of life (Gran et al., 2010, p. 29).

In addition, older people's family members as well as some healthcare staff recognized the effectiveness of non-pharmacological methods such as lying down in a quiet room, relaxation, physical therapy and exercise, and sweet talking for pain relief (Mentes et al., 2004).

Family member: “Face is more relaxed …. moves better…. is more talkative and would sleep better” (Mentes et al., 2004, p. 121).

Staff: “Comfort him, elevate his arm, try to reposition him, talk to him sweetly. I sometimes reposition them. I always talk softly to them to calm them down. It is the nurse's decision to give meds; sometimes I will insist if pain is bad or persistent” (Mentes et al., 2004, p. 123).

Expected pain

In contrast with the above-mentioned attitude, other older people considered pain as something expected and an unavoidable part of old age that was difficult to control and took the perspective that it was difficult to care for an older person in pain. Phrases such as putting up with pain, toleration, coping with pain, and resigning to pain reveal how such individuals surrendered and adapted to pain (Gran et al., 2010; Gudmannsdottir & Halldorsdottir, 2009; Higgins, 2005; Mentes et al., 2004; Yates et al., 1995).

Well I'm hurting all the time but as I say I have learnt to live with it now I have to. As I am talking to you now my back is killing me. There's nothing I can do about it really I would [tell others] to learn to live with it I just take it as part of my life (Yates et al., 1995, p. 670).

I'm full of it [pain] … I just put up with it…. (Higgins, 2005, p. 373).

I have to lay here until I die, but I have accepted it (Gran et al., 2010, p. 29).

Furthermore, some older people's family members and healthcare staff made comments confirming the older people's acceptance of pain as an inextricable quality of old age (Clark et al., 2004; Mentes et al., 2004).

Family member: “Was pretty tough. Wouldn't say ‘ouch’ unless in agony” (Mentes et al., 2004, p. 121).

Staff: “It's the generation of people in nursing homes. Pain is an expected part of life, and they've adjusted to it and are reticent to talk about it” (Clark et al., 2004, p. 745).

Putting up with pain was accompanied by a lack of energy, disability, personal loss, loneliness and isolation, and a low quality of life. On the other hand, older people described pain tolerance as a sign of courage, a test of faith and higher spirituality, a way to preserve pride, and a form of punishment for sins (Clark et al., 2004; Gran et al., 2010; Gudmannsdottir & Halldorsdottir, 2009; Higgins, 2005).

You know I use painkillers, but I do not know whether they help or not, but I suppose I should take them (Gran et al., 2010, p. 28).

I feel it [the pain] is what I have got to suffer, sort of thing … My life from the last …. One thing after another. First one thing then another (Higgins, 2005, p. 378).

These older people were not optimistic about taking medication to relive pain nor did they resist it. They were primarily influenced by healthcare staff's attitudes towards medication and therefore had a fear of drug addiction (Clark et al., 2004; Gran et al., 2010; Gudmannsdottir & Halldorsdottir, 2009; Higgins, 2005; Yates et al., 1995).

I still hope and expect that the pain will go away, but I don't hold out much hope … You might take a certain amount of it away, but you won't take the whole lot away. No drugs can stop all the pain (Yates et al., 1995, p. 671).

I kept telling her about this ugly pain. She'd take no notice of me. She'd shrug her shoulders and say, “it was arthritis, you have to put up with it.” Until one day she came and she said, “How was my neck?” (Higgins, 2005, p. 374).

### Normalizing suffering

The central metaphor of this study was “normalizing suffering,” integrating the themes and subthemes of this meta-synthesis (Fig. 1). It means that all aspects of the experience of pain in the nursing home context such as identity of pain, recognition of pain, and management of pain were components of suffering in nursing homes. Pain was described as originating from physical sources due to old age but went beyond mere physical pain to encompass emotional suffering as a separate source or intensifier of physical pain. In addition to living in the nursing home and physical illnesses as sources of distress, iatrogenic pain also increased older people's physical suffering. Emotional distress was translated into pain, caused by deaths and other afflictions in nursing homes.

The overlap between physical pain and emotional suffering that were described made pain assessment very complicated. Moreover, older people wished that healthcare staff would not ignore their pain and instead would validate their pain and suffering. Low trust in well-known methods of pain assessment due to their limited accuracy for pain recognition led healthcare staff to apply a person-centered method of pain assessment based on observation of pain clues. This method helped prevent the older people's unrecognized pain and suffering. Some of the participant's ambivalent perspective on the efforts to reduce suffering from pain influenced the effectiveness of these pain relief strategies. The cornerstone of the active approach to pain reduction among older people was their belief in the possibility of living without pain using non-pharmacologic methods. In contrast with this group, other older people expected pain-related suffering and tried to tolerate it as a normal part of life or as a means of testing faith and achieving higher spirituality. Throughout the findings and descriptions of the metaphor, the study directly and indirectly revealed that pain was mixed with suffering and in many cases was considered equivalent to suffering. This sort of suffering was considered normal and an accepted part of old age in the nursing home context. This attitude was a result of the characteristics of old age and the attitudes of different people involved in pain detection and management. In other words, pain was a phenomenon necessarily experienced during old age, even in nursing homes despite the presence of professional healthcare. Therefore, complaints about pain would be undermined due to the notion of “normalizing suffering.”

## Discussion

In this meta-synthesis, the experiences and perspectives of older people themselves as well as those of their family members and caregivers were described under three themes and six subthemes to form an integrated picture of pain experiences and pain management in the nursing home context.

The metaphor “normalizing suffering” symbolized and illustrated the hidden and unheard aspects of pain management in the nursing home context from different people's perspectives. This concept emphasizes the socialization process that normalizes pain and demonstrates how pain can become a routine and significant health risk for older people in nursing homes. According to Eriksson (2006), healthcare providers must be sensitive to their patients’ symbolic language in order to understand their suffering. Investigating patients’ comments and experiences of suffering can reveal “unavoidable” and “avoidable” sources of suffering in order to help organize interventions in line with anticipation, detection, and mitigation of older people's suffering (Mylod & Lee, 2013).

According to our findings, chronic health conditions were one of the main sources of pain, but older people's emotional issues that stemmed from living in nursing homes were intertwined with physical pain and even could worsen it. This finding is in line with the notion of pain as a complex experience comprising physical, psychological, social, and spiritual components (Tse et al., 2012). On the other hand, physical pain can become unendurable and cause an individual to suffer (Eriksson, 2006). Therefore, being attentive to older people's pain and making them feel comfortable has the potential to improve their spirituality and hope and promote inner harmony and healing (Closs, Cash, Barr, & Briggs, 2005; Hall, Dodd, & Higginson, 2014; Touhy, 2001). Such an approach can also balance positive and negative contributions to quality of life in old age (King et al., 2012).

We found that pain assessment in the nursing home context encompassed uncertainties. Challenges in pain assessment for older people such as cultural attitudes about pain, communication barriers, and cognitive issues hindered application of pain assessment scales. Previous research has reported that pain assessment in nursing homes is problematic due to the prevalence of cognitive issues in such environments (Weiner, Peterson, Ladd, McConnell, & Keefe, 1999). While valid and reliable behavioral pain assessment tools for older people with dementia who cannot self-report pain exist, nurses nevertheless face many challenges to assess pain reliably using standardized pain assessment instruments (Closs et al., 2005; Liu, 2014).

In the included studies, relatives and caregivers made statements that they thought that older people were exaggerating their pain. Such an attitude may lead older people to feel that have been discarded by society and are not taken seriously (Gjerberg, Førde, Pedersen, & Bollig, 2010; Oosterveld-Vlug et al., 2014). To address this issue, guidelines specifying pain assessment through one-to-one supervision for residents has been recommended (McConigley, Toye, Goucke, & Kristjanson, 2008).

This study identified an individualistic and unique method of pain assessment that drew on observations of non-verbal clues and behavioral changes that could suggest the possibility that an older person was experiencing pain. This finding confirms the inadequacy of existing pain management methods (Cook, 1998). Similar to our findings, complaining, negativity, repetitive sentences and questions, constant requests for attention, and verbal aggression have been identified as signs of pain in older people (Husebo et al., 2014). Though older people's preferences and beliefs impact how they report pain, healthcare staff must be careful when they question the reliability of pain reported by older people who suffer from different levels of cognitive impairment (Jones et al., 2005; Monroe et al., 2014). As a practical strategy, a multimodal assessment of pain including a report from the healthcare provider, older people's self-report, and external pain cues can lead to more precise measurement and improved treatment of pain (Fisher et al., 2002; Smith, 2005).

In this study, some older people responded to pain from the perspective that pain could be avoided in old age. Therefore, such older people actively sought pain relief including non-pharmacologic pain relief strategies. This finding is consistent with the fact that older people's emotional conditions and attitudes are essential to adapting to illness and disability (De Guzman et al., 2012). While analgesic medications are prescribed routinely, they are likely often inadequate or inappropriately prescribed (Higgins et al., 2004). Therefore, a combination of meditation and non-pharmacological strategies such as heat, positioning, and prayer can provide short-term pain relief and help older people cope with difficult conditions in nursing homes (Blomqvist & Edberg, 2002; Wilkes, Cioffi, Fleming, & LeMiere, 2011).

In contrast, some older people tried to put up with pain and described it as an expected problem that was difficult to control. Tolerating pain and having no hope of relief are common among some older people, who may not pursue pain relief (Higgins et al., 2004). Some older people tend to do everything they remain able to do on their own and will not accept help until their personal resources are exhausted (Eloranta, Routasalo, & Arve, 2008). Such a behavior might be their insecurity about their own value and capabilities, which results in a reluctance to speak about pain (Falk, Wijk, Persson, & Falk, 2013). In addition to the frequent ineffectiveness of reporting pain, the fear of not being understood influences individuals’ decision whether to speak up about their pain (Blomqvist & Edberg, 2002; Weiner & Rudy, 2002). Such a tendency not to express pain reveals how participants hide their pain in order to get on with their lives and to prevent the risk of being marginalized by healthcare staff on whom they depend (Higgins et al., 2004).

The older people included in this study were not optimistic about medication's ability to relieve pain. In addition, they were influenced by caregivers’ attitudes about medication and were afraid of drug addiction. Previous work has posited that healthcare staff lack sufficient knowledge and skills to identify and support older people's physical and psychosocial needs (Eloranta et al., 2008). Therefore, staff exclude older people from pain management decisions (Liu, 2014). Deficiencies in older people's drug use mainly comprise overuse of drugs (Hall-Lord, Johansson, Schmidt, & Larsson, 2003). However, healthcare providers’ disinclination to prescribe drugs is primarily rooted in fears about pain medications’ side effects (Baier et al., 2004). Fears about drug overuse usually worsen the immutable nature of persistent pain (Weiner & Rudy, 2002). Healthcare staff must inform older people that taking medication is not a sign of weakness and describe the consequences of failing to relieve pain (Jones et al., 2005).

## Limitations

This meta-synthesis was developed based on data collected in original studies that were selected for covering the experiences of older people, including the perspectives of their family members as well as caregivers involved in pain management in the nursing home context. Therefore, this study's quality is largely a product of the quality of data collected in the original studies chosen for meta-synthesis. A strength of the current study was that all authors conducted their reviews independently and looked for disconfirming data. While the integration of different individual's perspectives and experiences has not altered the focus of this study, it produced a more complete picture of the study phenomenon. However, the authors believe that in the future, there must be more complementary studies on the phenomena of pain recognition and management in the nursing home context.

## Conclusions

The quality of pain management in the nursing home context is complicated by older people's attitudes and knowledge—as well as that of their relatives and healthcare professional—regarding the identification of and responses to pain. Following this meta-synthesis’ main metaphor or core, “normalizing suffering,” an effort should be made to alter perspectives about pain's unavoidability in old age among those involved in pain management.

The common attitude of pain's unavoidability in old age must also change in society as a whole, including among older persons themselves as well as among those who are responsible for acknowledging, detecting, and relieving pain. This study's findings emphasize that pain as a primary source of suffering can be relieved, provided that older people are encouraged to report their pain. In addition, healthcare staff must receive sufficient training to take a person-centered approach towards pain assessment and management that considers all its elements.

This meta-synthesis’ theoretical model represents a starting point for future studies (Bondas & Hall 2007a; Britten et al., 2002) that can bridge the existing knowledge gap and help design cultural and contextual strategies to curb this issue in the nursing home context.

This study recommends the following strategies to help prevent suffering related to pain in the nursing home context:

- While older people commonly experience long-term illnesses, it must be emphasized that pain is not a normal part of living in old age;
- Healthcare providers should pay sufficient attention to pain relief;
- Suffering related to care and lack of caring should be prevented, as pain may be increased due to suffering related to the threat to integrity and losses of health, freedom, and independence;
- Older people's pain should be taken seriously and relieved as much as possible to avoid damaging their feeling of safety and quality of life in nursing homes;
- Standardized methods of pain assessment should be accompanied by observation of verbal and non-verbal clues to detect pain and to determine what may relieve pain, considering the frequent hidden messages of the individual older person.
